# Importance of nonsynonymous *OCA2* variants in human eye color prediction

**DOI:** 10.1002/mgg3.213

**Published:** 2016-03-11

**Authors:** Jeppe D. Andersen, Carlotta Pietroni, Peter Johansen, Mikkel M. Andersen, Vania Pereira, Claus Børsting, Niels Morling

**Affiliations:** ^1^ Section of Forensic GeneticsDepartment of Forensic MedicineFaculty of Health and Medical SciencesUniversity of CopenhagenDK‐2100CopenhagenDenmark; ^2^ Department of Mathematical SciencesAalborg UniversityDK‐9000AalborgDenmark

**Keywords:** Eye color, forensic DNA phenotyping, nonsynonymous variants, *OCA2*, pigmentation, rs74653330 and rs121918166, skin color

## Abstract

**Background:**

The color of the eyes is one of the most prominent phenotypes in humans and it is often used to describe the appearance of an individual. The intensity of pigmentation in the iris is strongly associated with one single‐nucleotide polymorphism (SNP), rs12913832:A>G that is located in the promotor region of *OCA2* (OMIM #611409). Nevertheless, many eye colors cannot be explained by only considering rs12913832:A>G.

**Methods:**

In this study, we searched for additional variants in *OCA2* to explain human eye color by sequencing a 500 kbp region, encompassing *OCA2* and its promotor region.

**Results:**

We identified three nonsynonymous *OCA2* variants as important for eye color, including rs1800407:G>A (p.Arg419Gln) and two variants, rs74653330:A>T (p.Ala481Thr) and rs121918166:G>A (p.Val443Ile), not previously described as important for eye color variation. It was shown that estimated haplotypes consisting of four variants (rs12913832:A>G, rs1800407:G>A (p.Arg419Gln), rs74653330:A>T (p.Ala481Thr), and rs121918166:G>A (p.Val443Ile)) explained 75.6% (adjusted *R* ^2^ = 0.76) of normal eye color variation, whereas rs12913832:A>G alone explained 68.8% (adjusted *R* ^2^ = 0.69). Moreover, rs74653330:A>T (p.Ala481Thr) and rs121918166:G>A (p.Val443Ile) had a measurable effect on quantitative skin color (*P *= 0.008).

**Conclusion:**

Our data showed that rs74653330:A>T (p.Ala481Thr) and rs121918166:G>A (p.Val443Ile) have a measurable effect on normal pigmentation variation.

## Introduction

The eye colors of humans were for a long time considered as a simple Mendelian trait with the brown eye color allele dominating the blue eye color allele. However, it is apparent that some humans have eye colors that are neither blue nor brown, but are perceived as green, gray, hazel, or different shades of these colors. Close‐up photographs of irides that appear intermediate in color (nonblue and nonbrown) show that some areas of the iris are blue while some areas are brown. A certain combination of blue and brown colors in the eye may appear green or hazel from a distance even though no such pigment exists.

The distinction between blue and brown eye colors can for the most part be explained genetically by one single‐nucleotide polymorphim (SNP), rs12913832:A>G in the hect domain and RCC1‐like domain 2 (*HERC2;* OMIM #605837) gene (Eiberg et al. 2008; Sturm et al. 2008). Although, rs12913832:A>G is located in intron 86 of *HERC2*, rs12913832:A>G is positioned in an enhancer element that regulates expression of the oculocutaneous albinism type II (*OCA2;* OMIM #611409) gene (Visser et al. 2012). It was shown that *OCA2* expression was reduced in lightly pigmented melanocytes with the derived allele rs12913832:G compared to darkly pigmented melanocytes with the ancestral allele rs12913832:A. According to the dominant hypothesis, brown eye color is the outcome in individuals with the genotype rs12913832:AA or rs12913832:GA. However, this is often not the case for individuals genotyped as rs12913832:GA. These individuals may have intermediate or even blue eye colors (Andersen et al. 2013). Furthermore, individuals with the rs12913832:GG genotype may have brown eyes (Andersen et al. 2013). Other variants, located in one of the exons or the promotor region of *OCA2,* were previously suggested to influence eye colors (Kayser et al. 2008; Duffy et al. 2007; Sulem et al. 2007; Mengel‐From et al. 2010). It was hypothesized that the derived allele of the nonsynonymous mutation rs1800407:A (p.419Gln) in *OCA2,* decreased the pigmentation level of the iris when found in cis phase with rs12913832:A (Andersen et al. 2013). An epistatic effect of the combination of rs1800407:G>A (p.Arg419Gln) and rs12913832:A>G was reported to increase the prediction accuracy of intermediate eye colors (Pospiech et al. 2014). Furthermore, a small increase in the prediction accuracy of intermediate eye colors was observed by also considering rs1129038:G>A that is in strong linkage disequilibrium with rs12913832:A>G in *HERC2* (Ruiz et al. 2013). Empirically estimated haplotypes of rs12913832:A>G, rs1129038:G>A, rs1800407:G>A (p.Arg419Gln), and the synonymous *HERC2* variation rs11636232:G>A (p.Gln3989Gln) indicated that multiple variants in the *HERC2*‐*OCA2* region influenced the eye color and that haplotype information in this region would be required to maximize eye color prediction (Mengel‐From et al. 2010).

Other pigmentary genes were shown to be associated with eye color, including tyrosinase (*TYR,* OMIM #606933), solute carrier family 45, member 2 (*SLC45A2*, OMIM #606202), solute carrier family 24, member 5, (*SLC24A5*, OMIM #609802) and interferon regulatory factor 4 (*IRF4*, OMIM #601900). Together with *HERC2*:rs12913832:A>G and *OCA2*:rs1800407:G>A (p.Arg419Gln), one SNP in each of these genes were proposed to be the best predictors for blue, brown and intermediate eye colors (Liu et al. 2009). An assay known as the IrisPlex was developed for eye color prediction (Walsh et al. 2010). The six SNPs have high prediction accuracy for blue and brown eye colors, but low accuracy for prediction of intermediate eye colors (Chaitanya et al. 2014). The prediction was largely driven by the predictive properties of rs12913832:A>G. Therefore, it was suggested that the prediction of eye colors using the IrisPlex assay should be restricted to rs12913832:A>G (Pietroni et al. 2014).

With the current consensus of three eye color categories (blue, intermediate, and brown), it may be difficult to find new markers for eye color due to the color variation within each eye color group. Moreover, individuals perceive eye colors differently. Self‐ or expert‐reported categorization of eye colors involves a large subjective element of individual assessment. This is a challenge when the investigated loci contribute with a small effect and the within variation in for example the intermediate eye color category may be larger than the effect of the investigated loci. This challenge can be overcome by using a quantitative measurement for the eye color phenotype (Andersen et al. 2013).

In this study, we investigated the genomic region encompassing *OCA2* (NM_000272) and its promotor (hg19, chr15: 28,000,023‐28,500,021) in relation to quantitatively measured eye colors. A total of 47 samples were sequenced using massive parallel sequencing. Of the 47 samples, 35 samples did not follow the dominant hypothesis based on the genotype of rs12913832:A>G. The samples included (1) subjects with light eye colors and the genotype rs12913832:GA and (2) subjects with dark eye colors and the genotype rs12913832:GG. Eight selected variants were genotyped in follow‐up studies of 515 Scandinavian samples and two southern European populations (217 Italians and 263 Portuguese).

## Materials and Methods

### Ethical compliance

The study was approved by the Danish Ethical Committee (H‐3‐2012‐023) and the Ethical Committee of University of Porto (No. 03/CEUP/2014). All participants gave signed informed consent. The samples were anonymized.

### Individuals and DNA purification

Blood samples from 263 unrelated Portuguese individuals were collected at the Cooperativa de Ensino Superior Politécnico e Universitário (CESPU), Porto, Portugal and University of Porto, Portugal. Furthermore, 562 Scandinavian samples and 217 Italian samples from two previous studies (Pietroni et al. 2014; Andersen et al. 2013) were used. DNA was purified from blood on FTA cards, using the BioRobot^®^ EZ1 Workstation (Qiagen, Venlo, Netherlands) and the EZ1 DNA Investigator Kit (Qiagen).

### Digital photographs and quantitative eye color

Photographs were taken at a distance of approximately 10 cm in “Raw” format with a Canon EOS 5D Mark V with ISO 800, shutter 1/100 and AV 18 using a Canon EF 100 mm f/2.8 L IS USM Macro Lens with manual focus. The white balance of “Raw” format photographs was changed to “Flash” using the Picture style editor software (Canon, Tokyo, Japan).

For each individual eye photograph, the eye color was determined quantitatively, using the Pixel Index of the Eye (PIE)‐score (Andersen et al. 2013).

### Quantitative measurements of skin color

Quantitative skin color measurements were performed, using a UV‐Optimize Scientific 555 (Chromo Light APS) (Kongshoj et al. 2006). The pigment protection factor (PPF) was employed as a measure for skin color. The PPF is a value for the protection against UVR provided by skin pigmentation and the top layer of epidermis (stratum corneum). Measurements were performed in triplicates on the buttock for each participant. The medians of the PPF triplicates were used for statistical analyses. All measured skin areas were free from nevi, freckles, tattoos, and hair. The instrument was calibrated with a white standard (ISO 2469).

### Sequencing of the OCA2‐HERC2 region

SureDesign (Agilent Technologies, Santa Clara, CA) was used to design capture‐probes for a 500 kbp region on chromosome 15 (hg19, chr15: 28,000,023‐28,500,021) for the Haloplex Target Enrichment kit (Agilent Technologies) with read length of 150 bp. The design included 11,272 amplicons targeting 471,380 bp. The library preparation was carried out according to the Haloplex Target Enrichment System version D. 5 protocol. All samples were sequenced on an Illumina MiSeq (Illumina, San Diego, CA) according to the manufacturer's instructions with paired‐end sequencing (2 × 150 bp) using the MiSeq Reagent Kit V2 (300 cycles).

### Analysis of sequence data

Illumina adaptors were trimmed using Flexbar (Dodt et al. 2012) with the following settings: minimum 6 bp overlap between adapter and read sequence allowing 2 bp mismatches, trimming of 3′end until base quality of Phred‐score 25, and a minimum read length of 30. Trimmed fastq‐files were aligned to the human reference sequence assembly Feb.2009 GRCh37/hg19 (UCSC Genome Browser) with the Burrows‐Wheeler Aligner (BWA)‐MEM algorithm (Li and Durbin 2009) to generate BAM‐files. The following settings were used with the BWA‐MEM algorithm: Mismatch penalty of 20 and gap penalty of 4. Variant Caller Files (VCFs) were generated using HaplotypeCaller of GATK ver. 2.6.5 (McKenna et al. 2010) with a minimum emission confidence threshold of Phred‐score 10 and a calling confidence threshold of Phred‐score 30. Postvariant analysis on VCFs was carried out in the statistical software R (R core team, version 3.1.1, URL http://www.R-project.org). Variants were accepted if they had a minimum coverage of 25 and heterozygote variants calls were accepted if the read frequency of the minor variant was >0.15. Accepted variants were analyzed using Alamut Batch (Interactive Biosoftware, Rouen, France).

### Variant typing

Eight variants, rs1800414:A>G (p.His615Arg), rs121918166:G>A (p.Val443Ile), rs74653330:A>T (p.Ala481Thr), rs1800407:G>A (p.Arg419Gln), rs1800401:C>T (p.Arg305Trp), rs12913832:A>G, rs62008729:C>T, and rs8030709:C>T were typed in a single PCR multiplex (Table S1). Samples were typed using the iPLEX1 Gold Kit (Agena Bioscience, San Diego, CA) in a final reaction volume of 6 *μ*L. The PCR contained 2 *μ*L DNA, 0.5 *μ*L 10× Buffer, 0.8 *μ*L 25 mmol/L MgCl_2_, 0.1 mL 25 mmol/L dNTP mix, 1.3 *μ*L 0.5 mmol/L primer mix (DNA Technology, Aarhus, Denmark), 0.2 *μ*L 5 U/*μ*L HotStarTaq, and 1.1 *μ*L H_2_O. The PCR was performed with the following conditions: denaturation at 94°C for 2 min followed by 45 cycles of 94°C for 20 sec, 60°C for 30 sec, 72°C for 1 min, followed by 72°C for 3 min. PCR products were treated with Shrimp Alkaline Phosphatase (SAP) (Agena Bioscience) at 37°C for 40 min and 85°C for 5 min. The single base extension (SBE) reaction contained 8 *μ*L SAP‐treated PCR products and 2 *μ*L iPLEX1 mix (Agena Bioscience). The iPLEX1 mix contained 0.2 *μ*L 10× iPLEX1 buffer, 0.2 *μ*L iPLEX1‐Termination mix, 0.94 *μ*L primer mix (DNA Technology), 0.04 *μ*L iPLEX1‐enzyme, and 0.62 *μ*L H_2_O. The SBE reaction was performed with the following conditions: Denaturation at 94°C for 30 sec followed by 40 cycles of 94°C for 5 sec, 52°C for 5 sec and 80°C 5 sec, 52°C for 5 sec and 80°C for 5 sec, 52°C for 5 sec and 80°C for 5 sec, 52°C for 5 sec and 80°C for 5 sec, 52°C for 5 sec and 80°C for 5 sec followed by 72°C for 3 min. A total of 40 *μ*L of molecular grade water and ion exchange resin (Agena Bioscience) was added to each sample. Samples were rotated for approximately 4 h and kept in the refrigerator for up to 4 days before spotting. Samples were spotted using a RS1000 Nanospotter (Agena Bioscience) and visualized on the MassARRAY1 Analyzer 4 System (Agena Bioscience) using the autorun settings. The six IrisPlex SNPs (rs12913832:A>G, rs1800407:G>A (p.Arg419Gln), rs12203592:C>T, rs1393350:G>A, rs12896399:G>T, and rs16891982:C>G (p.Phe374Leu)) were typed as part of a multiplex assay with 32 pigmentary SNPs using the iPLEX^®^ Gold kit (Agena Bioscience) as previously described (Andersen et al. 2013).

### Statistical analyses

Statistical calculations were carried out in R ver. 3.0.1. Haplotypes were estimated using PHASE ver. 2.1 (Stephens et al. 2001) with standard settings.

## Results

### Selection of individuals for sequencing

**Figure 1:**
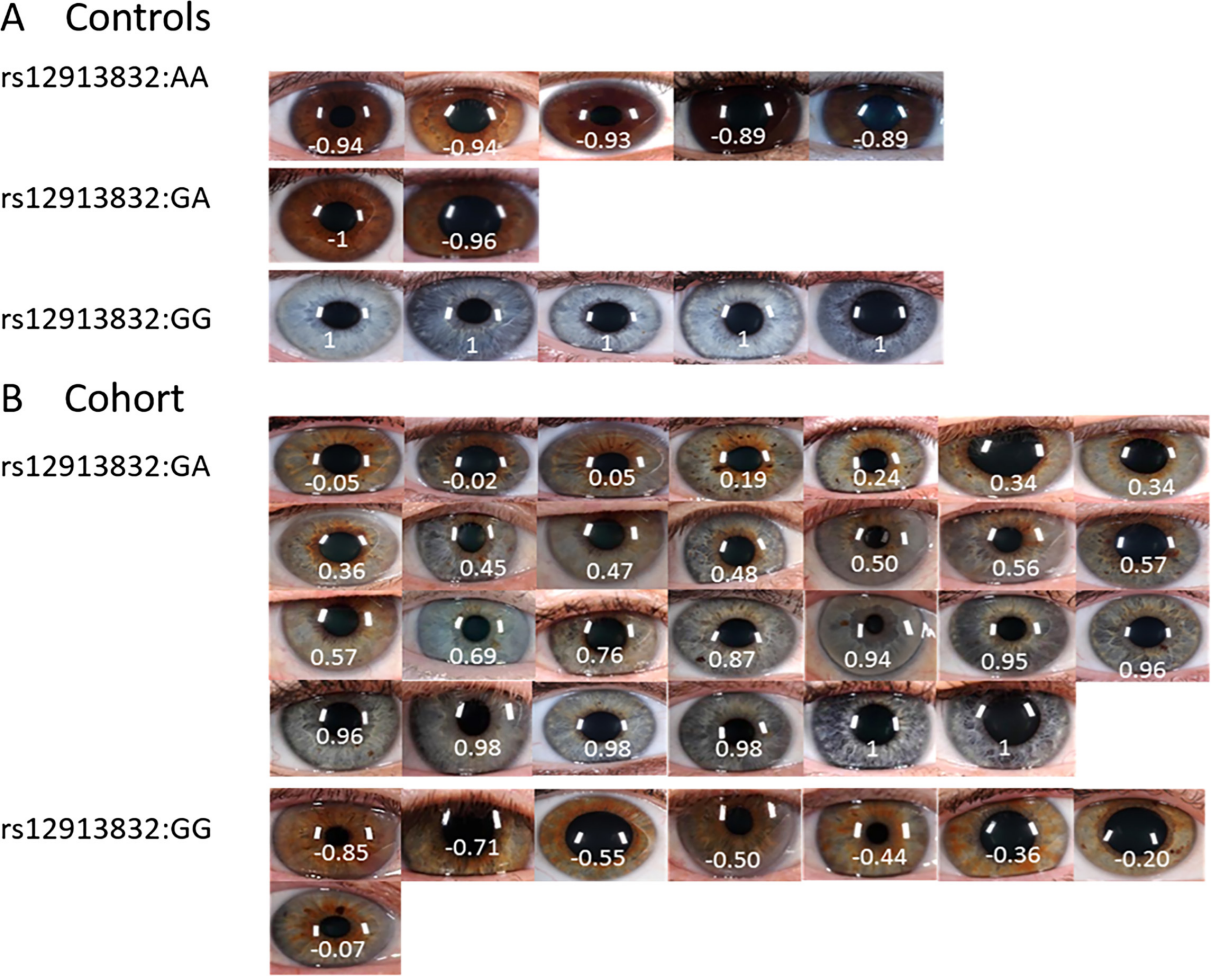
Digital eye images of the 47 individuals selected for sequencing. (A) Controls with corresponding rs12913832:A>G genotype and PIE‐scores. (B) Cohort that did not follow the dominant hypothesis based of the genotype of rs12913832 with corresponding rs12913832 genotype and PIE‐scores.

A total of 47 samples were selected for sequencing from an eye color database with digital eye images of more than 600 individuals of Scandinavian ancestry (Andersen et al. 2013). All individuals, including the 47 samples selected for sequencing, were genotyped for *HERC2*:12913832:A>G in a previous study (Andersen et al. 2013). Figure 1 shows the digital images of the 47 individuals and the corresponding PIE‐scores. The individuals included 35 individuals of Scandinavian ancestry that did not follow the dominant hypothesis determined by rs12913832:A>G and 12 individuals that followed the dominant eye color hypothesis. A total of 29 individuals were typed as rs12913832:GA. Of these, 27 did not follow the dominant hypothesis of rs12913832:A>G and had nonbrown eye colors (PIE‐scores from 1 to −0.05). Eight individuals were typed as rs12913832:GG but had nonblue eyes (PIE‐scores from −0.05 to −1). The remaining 12 samples were considered as controls. They included two individuals of the type rs12913832:GA with brown eyes (PIE‐scores of −0.96 and −1), five individuals typed as rs12913832:AA with brown eyes (PIE‐scores from −0.89 to −0.94), and five individuals typed as rs12913832:GG with blue eyes (PIE‐score = 1). The purpose of the sequencing experiment was to screen for new variants that may explain the variation in human eye color.

### Sequencing of the HERC2‐OCA2 region and discovery of candidate variants

A region of 500 kbp encompassing *OCA2* (NM_000272) and its promotor (hg19, chr15: 28,000,023‐28,500,021) was sequenced. The samples showed a median coverage ranging from 62 to 422 across the region. Positions with coverage below 25 were not considered for further analysis. The number of variant loci ranged from 193 to 752 in the sequenced individuals (Table S1). A total of 2,167 variant loci were found. Of these, 1,548 were known variants and 618 were novel variants (See Table S2). The SNP‐types from the previous study (Andersen et al. 2013), including the genotypes of rs12913832:A>G and rs1800407:G>A (p.Arg419Gln), were confirmed in all 47 samples. Five nonsynonymous variants rs1800414:A>G (p.His615Arg), rs74653330:A>T (p.Ala481Thr), rs121918166:G>A (p.Val443Ile), rs1800407:G>A (p.Arg419Gln), and rs1800401:C>T (p.Arg305Trp) were identified. Of the 27 nonbrown eyed individuals that were genotyped as rs12913832:GA, 19 (70%) individuals had one nonsynonymous variant allele of either rs1800407:A (p.419Gln), rs74653330:T (p.481Thr), or rs121918166:A (p.443Ile).

### Association between population‐specific OCA2‐HERC2 haplotypes and eye colors

**Figure 2:**
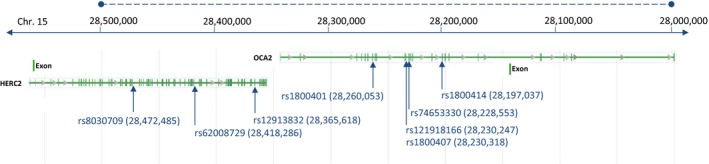
Chromosomal position of the eight candidate variants. Solid blue line indicates the chromosomal position. The dashed blue line represents the sequenced region (hg19, chr.15: 28,000,000‐28,500,000). Green lines represent gene and exons of *HERC2* (NM_004667) and *OCA2* (NM_000272). The arrows show the chromosomal position of the eight candidate variants.

The five nonsynonymous variants rs1800414:A>G (p.His615Arg), rs74653330:A>T (p.Ala481Thr), rs121918166:G>A (p.Val443Ile), rs1800407:G>A (p.Arg419Gln), and rs1800401:C>T (p.Arg305Trp) as well as the three variants (rs12913832:A>G, rs62008729:C>T, and rs8030709:C>T) located in the promotor region of *OCA2* (NM_000272) were selected as possible candidates to explain eye color variation (Fig. 2). The eight variants were typed in three European populations (515 Scandinavians, 217 Italians, and 263 Portuguese). All individuals had two parents of the same ancestry.

The most important determinant of eye color, rs12913832:A>G, had different allele frequencies in the three populations (Table 1). In the Scandinavian population, the frequency of the rs12913832:G was 86.8%, whereas the frequency was lower in the Italian (30.9%) and in the Portuguese populations (37.4%). In contrast, the allele rs1800407:A (p.419Gln) showed higher frequencies in the Italian (9.7%) and Portuguese (7.5%) populations compared to that in the Scandinavian population (4.7%). The three rare variant alleles, rs1800414:G (p.615Arg), rs74653330:T (p.481Thr), and rs121918166:A (p.443Ile), were only observed in the Scandinavian population.

**Table 1:** Minor allele frequencies of *HERC2‐OCA2* variants

	Frequencies		
	Scandinavians (*N = *562)	Italians (*N = *217)	Portuguese (*N = *263)
rs1800414:C	0.001	0.000	0.000
rs74653330:T	0.005	0.000	0.000
rs121918166:T	0.005	0.000	0.000
rs1800407:T	0.047	0.097	0.075
rs1800401:A	0.030	0.076	0.075
rs12913832:G	0.868	0.309	0.374
rs62008729:T	0.098	0.040	0.036
rs8030709:T	0.041	0.144	0.181

For statistical association of the variants with eye color, haplotypes were estimated, using PHASE ver. 2.1 (Stephens et al. 2001). Haplotypes were estimated based on the genotypes of rs74653330:C>T (p.Ala481Thr), rs121918166:G>A (p.Val443Ile), rs1800407:G>A (p.Arg419Gln), and rs12913832:A>G. Four variants, rs1800414:A>G (p.His615Arg), rs1800401:C>T (p.Arg305Trp), rs62008729:C>T, and rs8030709:C>T were excluded from haplotype reconstruction because they had no effect on haplotype association with the PIE‐score (*P *> 0.05). The estimated haplotypes and the haplotype frequencies in the various populations are shown in Table 2. Five different haplotypes were estimated, the ancient haplotype *OCA2*:A1 (NM_00272[rs74653330:C (p.Ala481); rs121918166:A (p.Val443); rs1800407:G (p.Arg419); rs12913832:A]), haplotype *OCA2*:G (NM_00272[rs74653330:C (p.Ala481); rs121918166:A (p.Val443); rs1800407:G (p.Arg419); rs12913832:G]), haplotype *OCA2*:A2 (NM_00272[rs74653330:C (p.Ala481); rs121918166:A (p.Val443); rs1800407:A (p.419Gln); rs12913832:A]), haplotype *OCA2*:A3 (NM_00272[ rs74653330:T (p.481Thr); rs121918166:A (p.Val443); rs1800407 (p.Arg419); rs12913832:A]) and haplotype *OCA2*:A4 (NM_00272[rs74653330:C (p.Ala481); rs121918166:G (p.443Ile); rs1800407:A (p.Arg419); rs12913832:A]).

**Table 2:** Frequencies of estimated *HERC2‐OCA2* haplotypes

					Frequencies		
Haplotypes	rs74653330: C>T (p.Ala481Thr)	rs129118166:A>G (p.Val443Ile)	rs1800407: G>A (p.Arg419Gln)	rs12913832: A>G	Scandinavians (*N = *562)	Italians (*N = *217)	Portuguese (*N = *263)
Haplotype *OCA2*:G	p.Ala481	p.Val443	p.Arg419	rs12913832:G	0.862	0.484	0.375
Haplotype *OCA2*:A1	p.Ala481	p.Val443	p.Arg419	rs12913832:A	0.100	0.426	0.557
Haplotype *OCA2*:A2	p.Ala481	p.Val443	p.419Gln	rs12913832:A	0.028	0.090	0.068
Haplotype *OCA2*:A3	p.481Thr	p.Val443	p.Arg419	rs12913832:A	0.005	0.000	0.000
Haplotype *OCA2*:A4	p.Ala481	p.443Ile	p.Arg419	rs12913832:A	0.005	0.000	0.000

**Figure 3:**
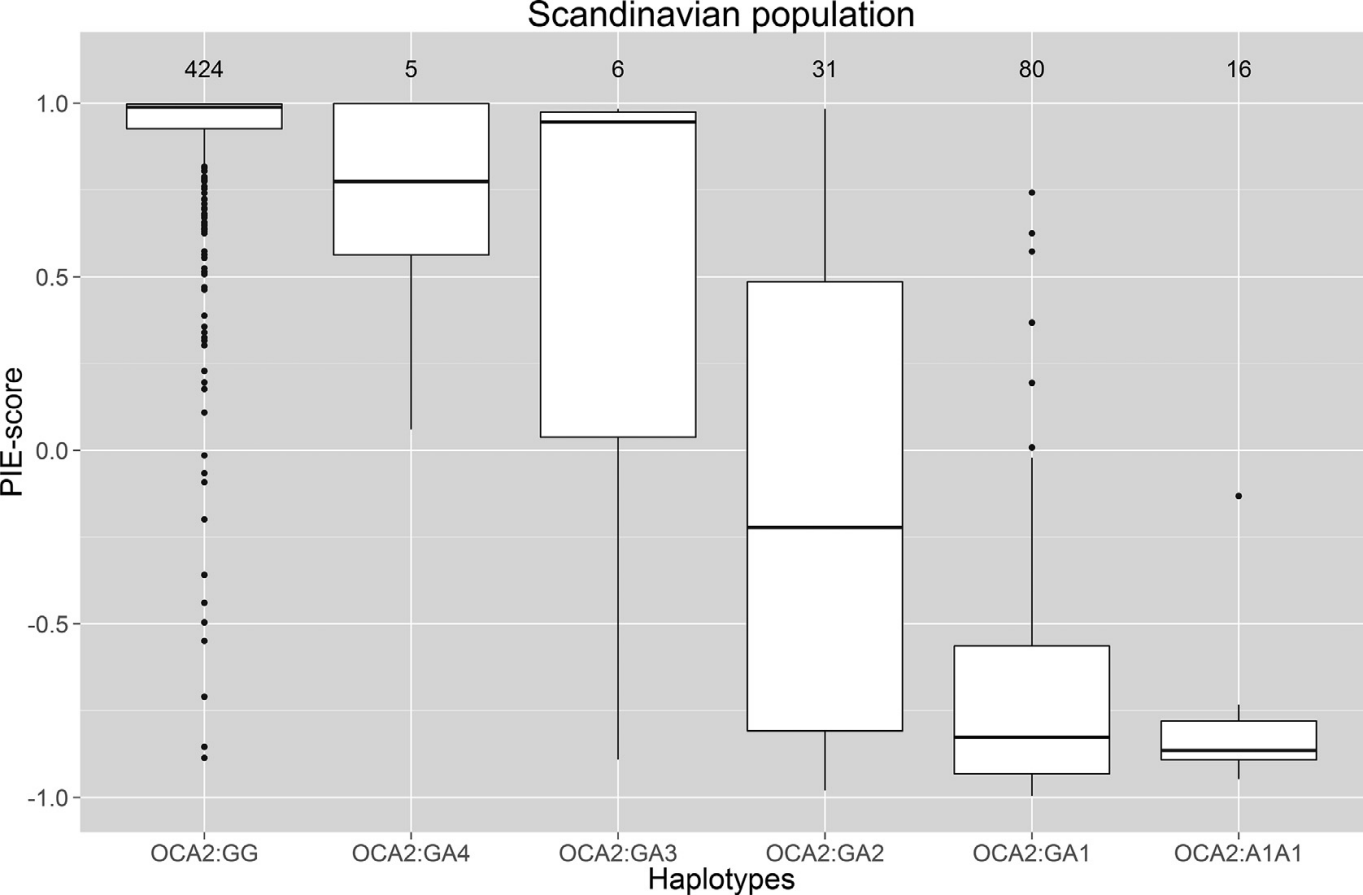
Estimated haplotypes and PIE‐scores in the Scandinavian population. The haplotypes were made from combinations of rs74653330 (p.Ala481Thr), rs121918166 (p.Val443Ile), rs1800407 (p.Arg419Gln), and rs12913832. Haplotype G (NM_00272[rs74653330 (p.Ala481); rs121918166 (p.Val443);rs1800407 (p.Arg419); rs12913832:G]), haplotype A1 (NM_00272[rs74653330 (p.Ala481); rs121918166 (p.Val443); rs1800407 (p.Arg419); rs12913832:A]), haplotype A2 (NM_00272[rs74653330 (p.Ala481); rs121918166 (p.Val443); rs1800407 (p.419:Gln); rs12913832:A]), haplotype A3 (NM_00272[rs74653330 (p.481Thr); rs121918166 (p.Val443); rs1800407 (p.Arg419); rs12913832:A]), and haplotype A4 (NM_00272[rs74653330 (p.Ala481); rs121918166 (p.443Ile); rs1800407 (p.Arg419); rs12913832:A]).

**Figure 4:**
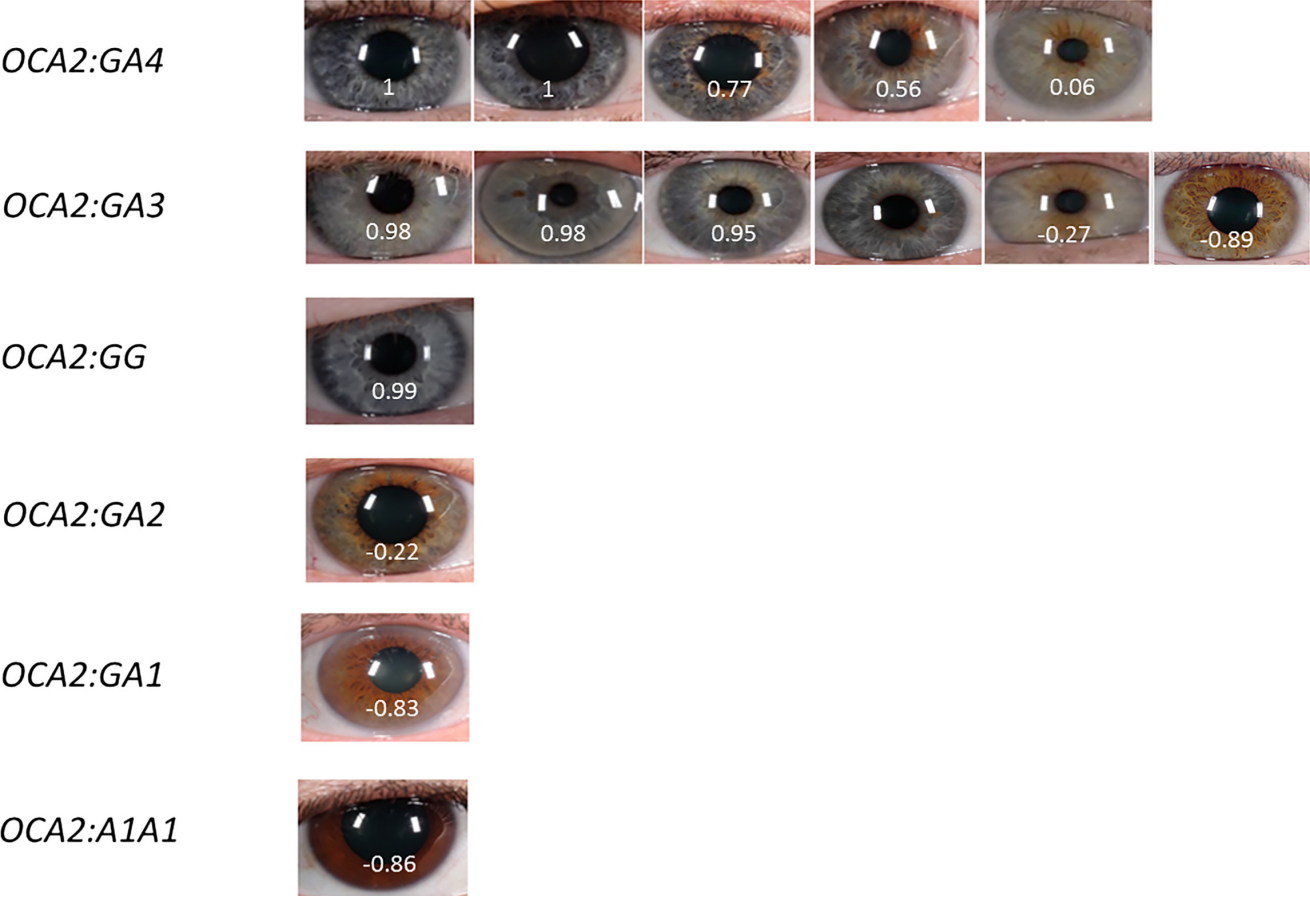
Digital eye images. Images from all individuals with *OCA2*:GA4 and *OCA2*:GA3 combinations are shown. Images with median PIE‐score values for the other haplotype combinations are shown. PIE‐scores are also given.

Figure 3 shows a boxplot of the six combinations of haplotypes and the PIE‐scores in the Scandinavian population. Only small variations in the PIE‐scores were observed in individuals that were homozygous for haplotype G (*OCA:GG*) or homozygous for haplotype A1 (*OCA2:A1A1*). Large variations were observed between individuals with the genotype rs12913832:GA (haplotype combination *OCA2*:GA4, *OCA2*:GA3, *OCA2*:GA2, and *OCA2*:GA1). A large effect on the PIE‐score was observed when either rs74653330:T (p.481Thr) or rs121918166:G (p.443Ile) were found in the same haplotype as rs12913832:A (haplotype *OCA2*:A3 and *OCA2*:A4). Individuals with either haplotype *OCA2*:A3 or *OCA2*:A4 in combination with the haplotype *OCA2*:G, had significantly (*P* < 1.5 × 10^−7^) lighter eye colors (*OCA2*:GA3 had a PIE‐score median of 0.95 while *OCA2*:GA4 had a PIE‐score median of 0.77) than individuals with the haplotype combinations *OCA2*:GA2 (PIE‐score median of −0.22) and *OCA2*:GA1 (PIE‐score median of −0.83). Figure 4 shows the digital eye images of individuals with *OCA2*:GA3 and *OCA2*:GA4 haplotype combinations. The haplotype with rs12913832:A and rs1800407:A (p.419Gln) (haplotype *OCA2*:A2) was associated with the PIE‐score. Individuals that were *OCA2*:GA2 had significantly lighter eye colors compared to *OCA2*:GA1 individuals (*P *= 5.7 × 10^−16^). A similar trend was found in the Italian and Portuguese populations in which *OCA2*:GA2 individuals had significantly lighter eye colors compared to *OCA2*:GA1 individuals (*P *= 0.01).

### The effect of HERC2‐OCA2 haplotypes on eye color in the Scandinavian population

Linear regression analyses were used to investigate the effect of the *HERC2‐OCA2* haplotypes on the PIE‐score, and hence, the eye color in the Scandinavian population. The effect of the haplotypes was found to be 76% (adjusted *R* ^2^ = 0.76). This effect was larger than the effect of the main predictor, rs12913832:A>G (adjusted *R* ^*2*^ **= 0.68) and also larger than the IrisPlex (rs12913832:A>G, rs1800407:G>A (p.Arg419Gln), rs12203592:C>T, rs1393350:G>A, rs12896399:G>T, and rs16891982:C>G (p.Phe374Leu)) (adjusted *R* ^*2*^ **= 0.71) (Table 3). To find the model with the highest correlation with the PIE‐score, we analyzed the predictive values of the *HERC2‐OCA2* haplotypes and the four additional IrisPlex markers (rs1393350:G>A, rs12203592:C>T, rs12896399:G>T, and rs16891982:C>G (p.Phe374Leu)). Backwards stepwise selection from the full model (*HERC2‐OCA2* haplotypes, rs1393350:G>A, rs12203592:C>T, rs12896399:G>T, and rs16891982:C>G (p.Phe374Leu)) was carried out using the adjusted *R* ^*2*^. The final model included only the *HERC2‐OCA2* haplotypes as predictors (adjusted *R* ^*2*^ = 0.76).

**Table 3:** Predictors for quantitative eye colors in the Scandinavian population

	Adjusted *R* ^*2*^
rs12913832:A>G	0.69
IrisPlex markers	0.71
*HERC2‐OCA2* haplotypes	0.76

### Association between HERC2‐OCA2 haplotypes and skin pigmentation in the Scandinavian population

**Figure 5:**
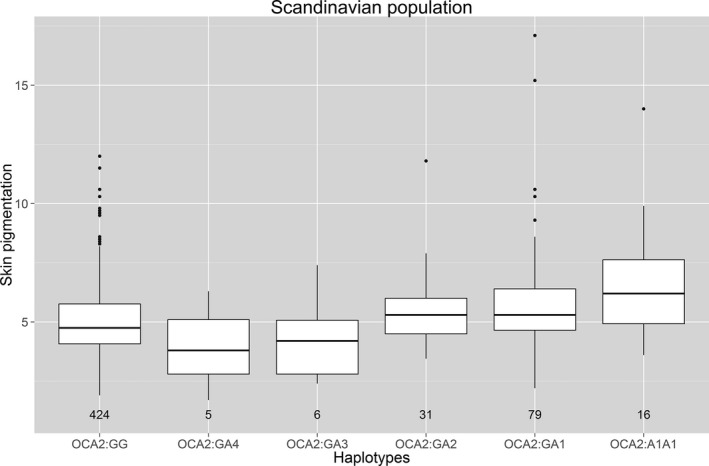
Estimated haplotypes and constitutive skin color in the Scandinavian population. The haplotypes were made from combinations of rs74653330 (p.Ala481Thr), rs129118166 (p.Val443Ile), rs1800407 (p.Arg419Gln) and rs12913832:A>G. Haplotype G (NM_00272[rs74653330 (p.Ala481); rs121918166 (p.Val443);rs1800407 (p.Arg419); rs12913832:G]), haplotype A1 (NM_00272[rs74653330 (p.Ala481); rs121918166 (p.Val443); rs1800407 (p.Arg419); rs12913832:A]), haplotype A2 (NM_00272[rs74653330 (p.Ala481); rs121918166 (p.Val443); rs1800407 (p.419:Gln); rs12913832:A]), haplotype A3 (NM_00272[rs74653330 (p.481Thr); rs121918166 (p.Val443); rs1800407 (p.Arg419); rs12913832:A]), and haplotype A4 (NM_00272[rs74653330 (p.Ala481); rs121918166 (p.443Ile); rs1800407 (p.Arg419); rs12913832:A]).

The association of *HERC2‐OCA2* haplotypes with normal skin pigmentation variation was investigated. The skin pigmentation was measured using reflectance measurements on the buttock area. Significant associations were observed between the haplotypes and levels of skin pigmentation (*P *= 2.7 × 10^−7^) (Fig. 5). Individuals with the *OCA2*:GA3 or *OCA2*:GA4 haplotype combinations had significantly lighter skin pigmentation than those homozygous for haplotype *OCA2*:G (*OCA2*:GG) (*P *= 0.008).

## Discussion

We sequenced a 500 kbp region spanning the *OCA2* gene and the *OCA2* promotor region in 47 individuals with various eye colors. The eye colors of 35 individuals were not in accordance with the hypothesis of a dominant genetic eye color model determined by rs12913832:A>G. Among these 35 individuals, 27 had the genotype rs12913832:GA and light eye colors. We found one of three nonsynonymous mutations, rs1800407:G>A (p.Arg419Gln), rs74653330:C>T (p.Ala481Thr), and rs121918166:A>G (p.Val443Ile) in 19 (70%) of the 27 individuals. The SNP rs1800407:G>A was previously suggested as a penetrance modifier of rs12913832:A>G (Sturm et al. 2008; Andersen et al. 2013). However, rs74653330:C>T (p.Ala481Thr) and rs121918166:A>G (p.Val443Ile) are, for the first time, demonstrated to be associated with normal eye color variation. The variant alleles, rs74653330:T (p.481Thr) and rs121918166:G (p.443Ile) were previously found in compound heterozygous state with other mutations in two Northern European individuals with characteristics of oculocutaneous albinism type II (Lee et al. 1994). The variant alleles, rs121918166:G (p.443Ile) was found together with rs121918167:T (p.743Leu) in an individual with typical characteristics of albinism. The variant allele, rs74653330:T (p.481Thr) was found together with a splice mutation, rs387906240:T (p.IVS17), in a patient with a mild clinical syndrome of oculocutaneous albinism type II. Subsequent studies reported rs74653330:T (p.481Thr) to be sporadically found in Japanese oculocutaneous albinism type II patients. Furthermore, rs74653330:T (p.481Thr) was reported to be an Asian‐specific hypopigmentation allele (Yuasa et al. 2007, 2011). However, the data presented here showed that rs74653330:C>T (p.Ala481Thr) also had an effect on pigmentation levels in Scandinavians. We found a significant effect on the eye color when the allele rs74653330:T (p.481Thr) or rs121918166:G (p.443Ile) were found in the same estimated haplotype such as rs12913832:A (haplotype *OCA2*:A3 and *OCA2*:A4). Individuals with the haplotype combination *OCA2*:GA3 or *OCA2*:GA4 had significantly lighter eye colors compared to those individuals with the haplotype combination *OCA2*:GA1, *OCA2*:GA2, and *OCA2*:A1. Furthermore, we found individuals with *OCA2*:GA3 or *OCA2*:GA4 to have the lightest skin color among all investigated individuals. The effect of *OCA2*:GA3 and *OCA2*:GA4 on eye and skin color strongly suggests that rs74653330:C>T (p.Ala481Thr) and rs121918166:A>G (p.Val443Ile) act as penetrance modifiers of rs12913832:A>G. It was previously shown that transfection of murine *Oca2‐*null melanocytes with a human wildtype *OCA2* cDNA construct restored melanin production. However, *Oca2‐*null melanocytes transfected with either human rs74653330:T (p.481Thr) or rs121918166:G (p.443Ile) cDNA constructs had significantly less melanin content than cells transfected with the human wildtype *OCA2* (Sviderskaya et al. 1997). This indicates that rs74653330:T (p.481Thr) and rs121918166:G (p.443Ile) variants results in dysfunctional OCA2 proteins that may explain the light eye and skin color of individuals with *OCA2*:GA3 and *OCA2*:GA4 haplotype combinations. OCA2 is a transmembrane protein, but the precise role of OCA2 is not completely understood. It was hypothesized that OCA2 regulates the pH of the melanosome and may be important for normal trafficking of tyrosinase, the key enzyme in melanin synthesis, to the melanosome (Raposo and Marks 2007). In vivo experiments of human melanocytes showed that dysfunctional OCA2 led to tyrosinase accumulation in the trans‐Golgi network (Toyofuku et al. 2002).

Our data showed that one allele of either rs74653330:T (p.481Thr) or rs121918166:G (p.443Ile) was sufficient to lower the pigmentation levels in healthy individuals. We did not observe any individuals with *OCA2*:A3A3, *OCA2*:A3A4 or *OCA2*:A4A4. These individuals are rare due to the low frequencies of rs74653330:T (p.481Thr) and rs121918166:G (p.443Ile).

Another nonsynonymous *OCA2* variant, rs1800407:G>A (p.Arg419Gln), also had an effect on the eye color in individuals with the rs12913832:GA genotype when rs1800407:A (p.419Gln) is in cis phase with rs12913832:A (haplotype *OCA2*:A2). The frequency of rs1800407:A (p.419Gln) is higher than rs74653330:T (p.481Thr) and rs121918166:G (p.443Ile). However, the effect of rs1800407:A (p.419Gln) on eye color was found to be smaller. Interestingly, rs74653330:T (p.481Thr) and rs121918166:G (p.443Ile) were only found in Scandinavians and not in the Italian and Portuguese populations. Frequencies from 1000 genomes phase 3 (http://browser.1000genomes.org) indicated that rs74653330:T (p.481Thr) is found in Native Americans (AMR) (0.4%), Europeans (EUR) (1.0%), and in even higher numbers in Asians (ASN) (2.7%), whereas rs121918166:G (p.443Ile) is only found in Europeans (EUR) (<1.0%). Together with the previous findings (Lee et al. 1994), our data suggests that rs121918166:G (p.443Ile) is mainly found in individuals of Scandinavian and Northern European descent.

Eight individuals with dark eyes and the genotype rs12913832:GG were also sequenced. We could not find any, single variant or variation patterns that could explain why these individuals did not have blue eyes. Our dataset of eight rs12913832:GG individuals with dark eyes is very small. A larger data set is required to find causative variants or variation patterns. We did not find any *OCA2* nonsynonymous variations in any of the eight individuals. This was expected as a nonsynonymous variation would most likely decrease the functionality of the OCA2 protein. We hypothesize that the unexpected dark eye color of these rs12913832:GG individuals is a product of induced *OCA2* expression. Somehow, the inhibitory effect of the rs12913832:G allele is reversed. This may include epigenetic regulation of the *OCA2* promotor. Different methylation patterns in blue and dark eyed individuals could explain the pigmentary difference. However, future studies are needed to clarify the regulation of *OCA2* expression in more detail.

To our knowledge, this study is the first to show that rs74653330:C>T (p.Ala481Thr) or rs121918166:A>G (p.Val443Ile) have a measurable effect on normal eye color variation. The effect is larger than that of rs1800407:G>A (p.Arg419Gln). Furthermore, it was shown that rs74653330:C>T (p.Ala481Thr) and rs121918166:A>G (p.Val443Ile) also have effects on normal skin color variation in Scandinavians. We suggest that rs74653330:T (p.481Thr) and rs121918166:G (p.443Ile) act as penetrance modifiers of rs12913832:A by lowering the pigmentation levels. To increase the prediction accuracy of existing prediction models for eye color, for example, the IrisPlex (Walsh et al. 2010) and *Snipper* (Ruiz et al. 2013), we suggest that rs121918166 and rs74653330 are included. These variants should also be considered in future prediction models for skin color.

## Conflict of Interest

None declared.

## Supporting information


**Table S1.** Median coverage, number of variant loci and alleles and PIE‐score for the 47 sequenced samples.

**Table S2.** Selected output from ALAMUT with information about the observed variants.

## References

[mgg3213-bib-0001] Andersen, J. D. , P. Johansen , S. Harder , S. R. Christoffersen , M. C. Delgado , S. T. Henriksen , et al. 2013 Genetic analyses of the human eye colours using a novel objective method for eye colour classification. Forensic Sci. Int. Genet. 7:508–515. 23948321 10.1016/j.fsigen.2013.05.003

[mgg3213-bib-0002] Chaitanya, L. , S. Walsh , J. D. Andersen , R. Ansell , K. Ballantyne , D. Ballard , et al. 2014 Collaborative EDNAP exercise on the IrisPlex system for DNA‐based prediction of human eye colour. Forensic Sci. Int. Genet. 11:241–251. 24880832 10.1016/j.fsigen.2014.04.006

[mgg3213-bib-0003] Dodt, M. , J. T. Roehr , R. Ahmed , and C. Dieterich . 2012 FLEXBAR–Flexible Barcode and adapter processing for next‐generation sequencing platforms. Biology. 1:895–905. 24832523 10.3390/biology1030895 PMC4009805

[mgg3213-bib-0004] Duffy, D. L. , G. W. Montgomery , W. Chen , Z. Z. Zhao , L. Le , M. R. James , et al. 2007 A three‐single‐nucleotide polymorphism haplotype in intron 1 of OCA2 explains most human eye‐color variation. Am. J. Hum. Genet. 80:241–252. 17236130 10.1086/510885 PMC1785344

[mgg3213-bib-0005] Eiberg, H. , J. Troelsen , M. Nielsen , A. Mikkelsen , J. Mengel‐From , K. W. Kjaer , et al. 2008 Blue eye color in humans may be caused by a perfectly associated founder mutation in a regulatory element located within the HERC2 gene inhibiting OCA2 expression. Hum. Genet. 123:177–187. 18172690 10.1007/s00439-007-0460-x

[mgg3213-bib-0006] Kayser, M. , F. Liu , A. C. Janssens , F. Rivadeneira , O. Lao , K. van Duijn , et al. 2008 Three genome‐wide association studies and a linkage analysis identify HERC2 as a human iris color gene. Am. J. Hum. Genet. 82:411–423. 18252221 10.1016/j.ajhg.2007.10.003 PMC2427174

[mgg3213-bib-0007] Kongshoj, B. , A. Thorleifsson , and H. C. Wulf . 2006 Pheomelanin and eumelanin in human skin determined by high‐performance liquid chromatography and its relation to in vivo reflectance measurements. Photodermatol. Photoimmunol. Photomed. 22:141–147. 16719868 10.1111/j.1600-0781.2006.00215.x

[mgg3213-bib-0008] Lee, S. T. , R. D. Nicholls , S. Bundey , R. Laxova , M. Musarella , and R. A. Spritz . 1994 Mutations of the P gene in oculocutaneous albinism, ocular albinism, and Prader‐Willi syndrome plus albinism. N. Engl. J. Med. 330:529–534. 8302318 10.1056/NEJM199402243300803

[mgg3213-bib-0009] Li, H. , and R. Durbin . 2009 Fast and accurate short read alignment with Burrows‐Wheeler transform. Bioinformatics 25:1754–1760. 19451168 10.1093/bioinformatics/btp324 PMC2705234

[mgg3213-bib-0010] Liu, F. , K. van Duijn , J. R. Vingerling , A. Hofman , A. G. Uitterlinden , A. C. Janssens , et al. 2009 Eye color and the prediction of complex phenotypes from genotypes. Curr. Biol. 19:R192–R193. 19278628 10.1016/j.cub.2009.01.027

[mgg3213-bib-0011] McKenna, A. , M. Hanna , E. Banks , A. Sivachenko , K. Cibulskis , A. Kernytsky , et al. 2010 The genome analysis toolkit: a MapReduce framework for analyzing next‐generation DNA sequencing data. Genome Res. 20:1297–1303. 20644199 10.1101/gr.107524.110 PMC2928508

[mgg3213-bib-0012] Mengel‐From, J. , C. Borsting , J. J. Sanchez , H. Eiberg , and N. Morling . 2010 Human eye colour and HERC2, OCA2 and MATP. Forensic Sci. Int. Genet. 4:323–328. 20457063 10.1016/j.fsigen.2009.12.004

[mgg3213-bib-0013] Pietroni, C. , J. D. Andersen , P. Johansen , M. M. Andersen , S. Harder , R. Paulsen , et al. 2014 The effect of gender on eye colour variation in European populations and an evaluation of the IrisPlex prediction model. Forensic Sci. Int. Genet. 11C:1–6. 24631691 10.1016/j.fsigen.2014.02.002

[mgg3213-bib-0014] Pospiech, E. , A. Wojas‐Pelc , S. Walsh , F. Liu , H. Maeda , T. Ishikawa , et al. 2014 The common occurrence of epistasis in the determination of human pigmentation and its impact on DNA‐based pigmentation phenotype prediction. Forensic Sci. Int. Genet. 11:64–72. 24681889 10.1016/j.fsigen.2014.01.012

[mgg3213-bib-0015] Raposo, G. , and M. S. Marks . 2007 Melanosomes–dark organelles enlighten endosomal membrane transport. Nat. Rev. Mol. Cell Biol. 8:786–797. 17878918 10.1038/nrm2258 PMC2786984

[mgg3213-bib-0016] Ruiz, Y. , C. Phillips , A. Gomez‐Tato , J. Alvarez‐Dios , C. M. de Casares , R. Cruz , et al. 2013 Further development of forensic eye color predictive tests. Forensic Sci. Int. Genet. 7:28–40. 22709892 10.1016/j.fsigen.2012.05.009

[mgg3213-bib-0017] Stephens, M. , N. J. Smith , and P. Donnelly . 2001 A new statistical method for haplotype reconstruction from population data. Am. J. Hum. Genet. 68:978–989. 11254454 10.1086/319501 PMC1275651

[mgg3213-bib-0018] Sturm, R. A. , D. L. Duffy , Z. Z. Zhao , F. P. Leite , M. S. Stark , N. K. Hayward , et al. 2008 A single SNP in an evolutionary conserved region within intron 86 of the HERC2 gene determines human blue‐brown eye color. Am. J. Hum. Genet. 82:424–431. 18252222 10.1016/j.ajhg.2007.11.005 PMC2427173

[mgg3213-bib-0019] Sulem, P. , D. F. Gudbjartsson , S. N. Stacey , A. Helgason , T. Rafnar , K. P. Magnusson , et al. 2007 Genetic determinants of hair, eye and skin pigmentation in Europeans. Nat. Genet. 39:1443–1452. 17952075 10.1038/ng.2007.13

[mgg3213-bib-0020] Sviderskaya, E. V. , D. C. Bennett , L. Ho , T. Bailin , S. T. Lee , and R. A. Spritz . 1997 Complementation of hypopigmentation in p‐mutant (pink‐eyed dilution) mouse melanocytes by normal human P cDNA, and defective complementation by OCA2 mutant sequences. J. Invest. Dermatol. 108:30–34. 8980282 10.1111/1523-1747.ep12285621

[mgg3213-bib-0021] Toyofuku, K. , J. C. Valencia , T. Kushimoto , G. E. Costin , V. M. Virador , W. D. Vieira , et al. 2002 The etiology of oculocutaneous albinism (OCA) type II: the pink protein modulates the processing and transport of tyrosinase. Pigment Cell Res. 15:217–224. 12028586 10.1034/j.1600-0749.2002.02007.x

[mgg3213-bib-0022] Visser, M. , M. Kayser , and R. J. Palstra . 2012 HERC2 rs12913832 modulates human pigmentation by attenuating chromatin‐loop formation between a long‐range enhancer and the OCA2 promoter. Genome Res. 22:446–455. 22234890 10.1101/gr.128652.111 PMC3290780

[mgg3213-bib-0023] Walsh, S. , F. Liu , K. N. Ballantyne , M. van Oven , O. Lao , and M. Kayser . 2010 IrisPlex: a sensitive DNA tool for accurate prediction of blue and brown eye colour in the absence of ancestry information. Forensic Sci. Int. 3:170–180. 10.1016/j.fsigen.2010.02.004 20457092

[mgg3213-bib-0024] Yuasa, I. , K. Umetsu , S. Harihara , A. Miyoshi , N. Saitou , K. S. Park , et al. 2007 OCA2 481Thr, a hypofunctional allele in pigmentation, is characteristic of northeastern Asian population. J. Hum. Genet. 52:690–693. 17568986 10.1007/s10038-007-0167-9

[mgg3213-bib-0025] Yuasa, I. , S. Harihara , F. Jin , H. Nishimukai , J. Fujihara , Y. Fukumori , et al. 2011 Distribution of OCA2 *481Thr and OCA2 *615Arg, associated with hypopigmentation, in several additional populations. Leg. Med. (Tokyo) 13:215–217. 21565543 10.1016/j.legalmed.2011.04.003

